# Cytosolic and Acrosomal pH Regulation in Mammalian Sperm

**DOI:** 10.3390/cells13100865

**Published:** 2024-05-17

**Authors:** Julio C. Chávez, Gabriela Carrasquel-Martínez, Sandra Hernández-Garduño, Arturo Matamoros Volante, Claudia L. Treviño, Takuya Nishigaki, Alberto Darszon

**Affiliations:** 1 Departamento de Genética del Desarrollo y Fisiología Molecular, Instituto de Biotecnología (IBT), Universidad Nacional Autónoma de México (UNAM), Cuernavaca 62210, Morelos, Mexico; julio.chavez@ibt.unam.mx (J.C.C.); gabycarrasquel@yahoo.com (G.C.-M.); 2 CITMER, Medicina Reproductiva, México City 11520, Mexico; 3 Departamento de Morfología, Facultad de Medicina Veterinaria y Zootecnia, Universidad Nacional Autónoma de México (UNAM), México City 04510, Mexico; sandrahernandez@fmvz.unam.mx; 4 Department of Electrical and Computer Engineering and School of Biomedical Engineering, Colorado State University, Fort Collins, CO 80523, USA; arturomv@colostate.edu

**Keywords:** mammalian sperm capacitation, acrosomal pH, proton channels and transporters, bicarbonate transport, cytosolic pH

## Abstract

As in most cells, intracellular pH regulation is fundamental for sperm physiology. Key sperm functions like swimming, maturation, and a unique exocytotic process, the acrosome reaction, necessary for gamete fusion, are deeply influenced by pH. Sperm pH regulation, both intracellularly and within organelles such as the acrosome, requires a coordinated interplay of various transporters and channels, ensuring that this cell is primed for fertilization. Consistent with the pivotal importance of pH regulation in mammalian sperm physiology, several of its unique transporters are dependent on cytosolic pH. Examples include the Ca^2+^ channel CatSper and the K^+^ channel Slo3. The absence of these channels leads to male infertility. This review outlines the main transport elements involved in pH regulation, including cytosolic and acrosomal pH, that participate in these complex functions. We present a glimpse of how these transporters are regulated and how distinct sets of them are orchestrated to allow sperm to fertilize the egg. Much research is needed to begin to envision the complete set of players and the choreography of how cytosolic and organellar pH are regulated in each sperm function.

## 1. Introduction

The proton (H^+^), a hydrogen atom stripped of its electron, is the smallest cation in the universe and one of the most controlled ions in the cytosol and in intracellular compartments. Its cologarithm, the pH, is challenging to regulate because: (1) H^+^ are rapidly released or consumed as acid-base equivalents in many reactions; (2) cells generate and control H^+^ gradients to store and transform free energy and to signal; (3) the availability of H^+^, thus pH, determines the surface charge of molecules, their physicochemical properties, and how they interact with each other [1]. Cellular processes operate within a narrow pH range, so precise regulation of pH is essential for cell function. As cells are compartmentalized, enzyme activity, gene expression, membrane channel operation, and transport are influenced by the pH of the cell compartment where the corresponding proteins are localized. The external pH (pHe) to which cells are exposed can vary and alter the cytosolic and intraorganellar pH [2]. Although mammalian sperm possess the nucleus, the redundant nuclear envelope (which contains residual nuclear material), and mitochondria as essential organelles, this review mainly focused on the regulation of the cytosolic pH (pHi) and the intra-acrosomal pH (pHa). Cell metabolic activity influences both pHi and pHa. Cells employ a set of proteins and transporters that move or transform acids and/or bases to finely regulate the pH in all compartments, since failure to do so has severe functional consequences. H^+^, HCO_3_^−^, and CO_2_ are stellar elements of pH regulation that are used not only to ensure proper cell function but also to participate in key signaling events necessary for organellar and cell-cell communication [3,4].

During sperm storage in the epididymis, the pHi is relatively acidic to avoid sperm activation and therefore unnecessary energy loss [5]. In their journey through the female genital tract, mammalian sperm undergo dramatic ion concentration changes in the media that surrounds them, which include H^+^, HCO_3_^−^, Na^+^, K^+^, and Ca^2+^ [6]. Sperm possess a unique set of ion channels and transporters, essential for their function, that are significantly pH-dependent. In this regard, pH regulation is extremely relevant for these cells. 

In the reproductive female tract, sperm must perform a maturational process to be able to fertilize the egg, called capacitation [7,8,9]. Capacitation is accompanied by changes in protein phosphorylation, membrane potential hyperpolarization, pHi and pHa alkalinization, and motility hyperactivation [7]. Sperm pHi alkalinization is a consequence of pHe and HCO_3_^−^ increases that occur as the cell enters and travels along the oviduct. For instance, external HCO_3_^−^ is elevated by the prostatic fluid [10]. The vaginal portion is acidic, the uterus is neutral, and the upper isthmus and ampulla (the site where egg fertilization occurs) are alkaline [11].

To respond to these changes in pHe and HCO_3_^−^, sperm specific ionic channels are modulated by pHi, like the Ca^2+^ channel CatSper [12], the K^+^ channel SLO3 [13,14,15], the Na^+^/H^+^ exchanger sNHE [16,17,18], and others such as the H^+^ channel Hv1 [19,20]. Regarding their physiological function briefly: CatSper activation modulates motility hyperactivation and possibly the acrosome reaction (AR) [21]. SLO3 is involved in the capacitation-associated membrane potential hyperpolarization, a change required before fertilization. In addition, sNHE modulates and maintains pHi and regulates the expression of sperm-specific soluble adenylate cyclase (sAC) [22]. sAC is modulated by Ca^2+^ and HCO_3_^−^ and is responsible for cyclic AMP production [23,24]. Moreover, pHi alkalinization modulates the sperm flagellar movement since the flagellar dynein ATPases are pHi-sensitive [25].

The regulation of sperm activity is closely linked to CO_2_/HCO_3_^−^ and pHi levels. As indicated earlier, several mechanisms are involved in sperm pHi regulation, and these mechanisms may vary across different species [26,27]. In the sections that follow, we will discuss in more detail the transporters that regulate pHi and pHa.

## 2. Ion Transporters That Regulate Cytosolic and Acrosomal pH

### 2.1. The CO_2_/HCO_3_^−^ Pair and Carbonic Anhydrases (CAs)

Both CO_2_/HCO_3_^−^, the primary physiological cell buffering system responsible for maintaining a pH balance, exhibit high mobility. Their acid-base equilibrium is governed by two pKa values (6.4 and 10.3), involving two additional chemical species difficult to track, H_2_CO_3_ and CO_2_. Additionally, CO_2_ is continually generated as the final product of aerobic cellular respiration. Furthermore, the equilibrium between gaseous CO_2_ and dissolved CO_2_ is influenced by factors like pH, temperature, and CO_2_ partial pressure [28]. 

Several H^+^ transporters contribute to pHi regulation, but the open nature of the CO_2_/HCO_3_^−^ system also aids this process. CO_2_ can easily diffuse through the plasma membrane, while HCO_3_^−^ is an impermeant anion, requiring transport proteins to enhance its movement across membranes [29]. Carbonic anhydrases (CAs) are ubiquitous metalloenzymes, relying on Zn^2+^ as a cofactor. They are found in both prokaryotes and eukaryotes and catalyze the reversible hydration of CO_2_ to HCO_3_^−^, while also possessing other catalytic activities [30]. CAs are encoded by eight gene families or classes identified with the Greek letters: α, β, γ, δ, ζ, η, θ, ι. These eight classes can be classified as non-homologous, isofunctional enzymes since they catalyze the same reaction even though they show low sequence similarity [31]. In mammals, there are 16 different α-CAs, named CAI through CAXVI. These enzymes have diverse tissue distributions and can be found in different subcellular compartments (soluble in the cytosol, membrane-bound with the enzymatic activity facing either the intracellular or extracellular side, mitochondrial, etc.) [30]. CAs play a crucial role in counterbalancing the slow kinetics of CO_2_/HCO_3_^−^ interconversion and establishing substrate gradients for transporter activity, enhancing their efficiency and speed in facilitating rapid pHi adjustments [31]. CAs and HCO_3_^−^ transporters may form a structural complex termed the HCO_3_^−^ transport metabolon [32]. In some instances, cytosolic CAII interacts with members of the Na^+^/H^+^ exchanger family like the NHE1 and members of the anion exchanger family like the AE1 [33] to form HCO_3_^−^ transport metabolons. This accelerates the transport rates of NHE1 and AE1 by increasing the local concentration of transport substrates. Similarly, CII also forms a physical interaction with the Cl^−^/HCO_3_^−^ exchanger SLC26A6, which is down-regulated by PKC phosphorylation, reducing the HCO_3_^−^ transport rate [32]. Moreover, an extracellular interaction between AE1 and CAIV forms the extracellular component of a HCO_3_^−^ transport metabolon [33].

**Figure 1:**
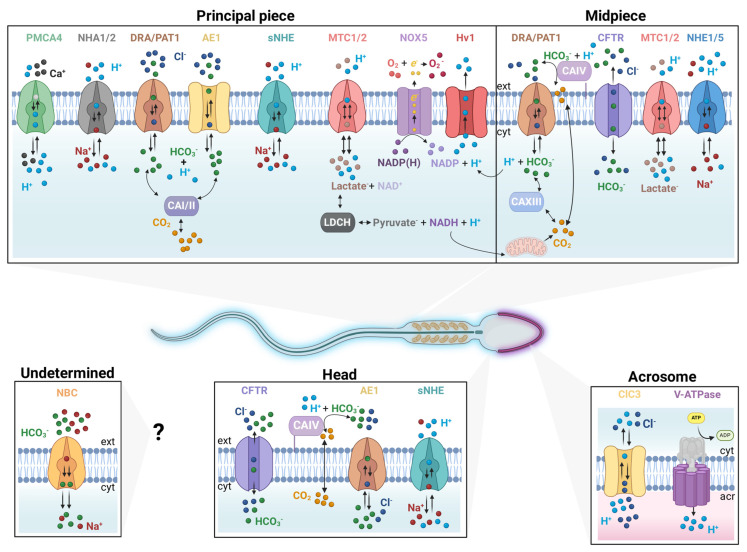
Schematic representation of the location of the proteins related to pHi regulation in mammalian sperm.

The presence of several CAs in mammalian sperm was reported using different experimental approaches. CAI, CAII, CAIV, and CAXIII were detected in human sperm mainly by immunological techniques [34,35,36,37,38]. CAI and CAII were localized in the principal piece of the flagellum, while CAIV and CAXII were found in the midpiece (Figure 1, Table 1). Importantly, the presence of the intracellular CAII and the extracellular CAIV in human sperm has been confirmed using a proteomic approach [39]. Additionally, these two enzymes were also reported to be present in mouse sperm [35,37]. Pharmacological approaches have illustrated the role of CAs in different sperm functions, such as completion of capacitation, control of motility, and hyperactivation, as well as in the AR [35,37]. However, the specificity of these drugs is not always unequivocal, and therefore, the precise CA involved in each process may be debatable. Interestingly, it is proposed that the extracellular CAIV gets transferred to sperm during epididymal transit [40], and the genetic elimination of this isoenzyme has revealed relevance in mouse sperm motility [37].

In a recent study, it was proposed that CAs may play a role in the regulation of human sperm pH by counteracting the acidification evoked by passive diffusion of CO_2_ [39].

### 2.2. HCO_3_^−^ Transporters

#### 2.2.1. Solute Carrier Family 4 (SLC4)

The SLC4 family comprises ten members, the major group of transporters carrying HCO_3_^−^ [70]. According to the mechanism, the family is classified into Na^+^-independent and Na^+^-dependent HCO_3_^−^ transporters. Na^+^-independent mechanisms involve an electroneutral anion exchange (AE) of Cl^−^/HCO_3_^−^, carried out by SLC4A1 (AE1), SLC4A2 (AE2), SLC4A3 (AE3), and two Na^+^-coupled Cl^−^/HCO_3_^−^ exchangers, SLC4A8 (NDCBE) and SLC4A9 (AE4). Na^+^-dependent HCO_3_^−^ transport comprises the electrogenic cotransporters SLC4A4 (NBCe1; stoichiometry 1Na^+^: 2HCO_3_^−^) and SLC4A5 (NBCe2; 1Na^+^: 3HCO_3_^−^), and the electroneutral cotransporters SLC4A7 (NBCn1; 1Na^+^: 1HCO_3_^−^) and SLC4A10 (NCBE or NBCn2) [70,71]. 

Regarding the expression of Na^+^-independent SLC4 transporters, immunolocalization experiments revealed AE1 is localized in the head and principal piece of human sperm (Figure 1). Due to the lack of specific inhibitors, the participation of this protein in sperm function has not been demonstrated. Recently, it was reported that AE1 undergoes tyrosine phosphorylation [43], a well-known protein regulating mechanism during sperm capacitation [72,73]. On the other hand, AE2 is present in the testis, epididymis, vas deferens, and sperm. Mice lacking the expression of this transporter present infertility problems due to a failure in spermatogenesis [44]. Unlike AE2 and AE1, AE3 has been detected only in Leydig cells and in developing germ cells [71]. 

For instance, the Na^+^-dependent SLC4 members NBCe1, NBCn1, and NBCe2 are found throughout the male reproductive tract. Their presence has been detected through techniques such as western blot, immunocytochemistry, and in situ hybridization [71]. The participation of an electrogenic Na^+^/HCO_3_^−^ cotransporter was proposed in human and mouse sperm [45,74]. The authors concluded that NBC acts as one of the first routes of HCO_3_^−^ transport required by the activations of HCO_3_^−^ signaling pathways, including the capacitation-associated hyperpolarization of the plasma membrane and tyrosine phosphorylation. Unfortunately, the identity and localization of this NBC are still unknown (Figure 1). But considering its electrogenic properties, the most likely candidates are NBCe1 or NBCe2. A recent study by Grahn et al. [39] suggests that the rise in sperm HCO_3_^−^ concentration may be due to CO_2_ diffusion rather than influx through specific transporters. The conversion of CO_2_ to HCO_3_^−^ generates H^+^, leading to pHi elevation. This is further supported by the lack of evidence for NBC expression using proteomic techniques [39].

#### 2.2.2. Solute Carrier Family 26 (SLC26)

The SL26 family of transporters comprises 10 members in mammals (SLC26A1 to SLC26A11; SLC26A10 being a pseudogene) [75]. SLC26 proteins act as anion exchangers and anion channels. They transport halides (Cl^−^, I^−^, Br^−^), thiocyanate (SCN^−^), monovalent oxyanions (OH^−^, HCO_3_^−^, NO_3_^−^, format, glyoxylate), and divalent oxyanions (SO_4_^2−^, oxalate) (Alper and Sharma, 2013). The stoichiometry has been only described for SLC26A3 (2Cl^−^: 1HCO_3_^−^) and for SLC26A6 (1Cl^−^/ 2HCO_3_^−^), both being electrogenic [76]. The activity of these proteins is co-regulated by the cystic fibrosis transmembrane conductance regulator channel (CFTR) through PDZ domain-containing scaffold proteins [46]. In addition to the PDZ interaction, the SLC26 transporters also possess a C-termini region called the “sulphate transporter anti-sigma factor antagonist domain” (STAS). The STAS domain interacts with the CFTR regulatory domain (R) through a cAMP-activated protein kinase (PKA) phosphorylation-dependent mechanism [77]. Interestingly, mammalian sperm PKA significantly participates in the regulation of several processes during capacitation, including pHi [50,51].

SLC26A3 (DRA) [46,48], SLC26A6 (PAT1) [46], and SLC26A8 (TAT1) [78,79] were detected by immunohistochemistry and Western blot techniques in mouse epididymal cells and sperm. PAT1 was found in the midpiece of mouse sperm, whereas TAT1 was localized in the annulus and equatorial segment (Figure 1) [46,78]. Moreover, a recent proteomic report indicated the presence of only DRA and TAT1 in human sperm [39]. DRA-deficient mice present health issues such as congenital diarrhea and reduced fertility [80]. While the TAT1 null mouse is infertile due to motility deficiencies since the flagellar structure is altered [78]. In addition, the absence of TAT1 in human sperm causes asthenozoospermia [81]. However, HCO_3_^−^ transport in TAT1 has not been reported, and its role in pHi is still unclear.

#### 2.2.3. The Cystic Fibrosis Transmembrane Conductance Regulator (CFTR)

CFTR acts as an ion channel permeable to Cl^−^, HCO_3_^−^, and other anions [82]. This protein is an ATP-gated channel regulated by PKA, as phosphorylation is essential for both channel opening and ATP association [83]. Function-impairing mutations in the CFTR gene have been associated with reduced fertility in both men and women. Men with cystic fibrosis are infertile due to a congenital bilateral absence of the vas deferens. Furthermore, mutations of CFTR occur with high frequency, suggesting its participation in other important events such as sperm capacitation [84].

CFTR expression has been reported using immunological tools in both mature human and mouse sperm. It is localized in the mid-piece and the equatorial segment of the head [53,85]. Moreover, Figueiras-Fierro et al. demonstrated CFTR activity in mature mouse sperm using electrophysiological techniques [86]. Heterozygous CFTR mutant mice showed lowered fertility rates [53]. Supporting this notion, the treatment of human and mouse sperm with a specific CFTR inhibitor decreased hyperactivation, AR, and the penetration of zona pellucida-free hamster eggs [49,53]. Similarly, inhibiting CFTR channels results in defective pHi alkalinization and hinders the activation of the cAMP/PKA pathway [50,51,74]. Intriguingly, despite this evidence, a recent proteomic study did not find CFTR expression in human sperm [39]. As mentioned in the previous section, such findings questioned the role of CFTR (and other HCO_3_^−^ transporters) not only as a mechanism of pHi regulation but also the well-accepted notion of HCO_3_^−^ influx on sperm capacitation. 

### 2.3. Na^+^/H^+^ Exchangers (NHEs) 

The NHEs are ubiquitous ion transporters found in most animal tissues [87,88]. These integral membrane proteins regulate pHi in an electroneutral manner, utilizing the energy stored in the inward Na^+^ gradient to export intracellular H^+^ [58]. NHEs belong to the solute carrier 9 (SLC9) transporter family [87]. This family of proteins comprises different isoforms grouped into three subfamilies (SLC9A, SLC9B, and SLC9C).

Mammalian testis/sperm expresses eight NHE protein isoforms. NHE1 (SLC9A1) and NHE5 (SLC9A5) are located in the midpiece of the human, mouse, and rat sperm flagellum [55,89]. sNHE/NHE11 (SLC9C2) is found in the heads of human and mouse sperm [58,90,91]. sNHE/NHE10 (SLC9C1), the sperm specific NHE, NHA1 (SLC9B1), and NHA2 (SLC9B2) are present in the principal piece of human and mouse sperm flagellum [16,18,22,56,57,92,93]. While the isoforms described above are supposed to be localized on the plasma membrane, NHE3 (SLC9A3) and NHE8 (SLC9A8) have been detected in the developing human and mouse sperm acrosome, respectively [90,94,95,96,97]. 

**Figure 2:**
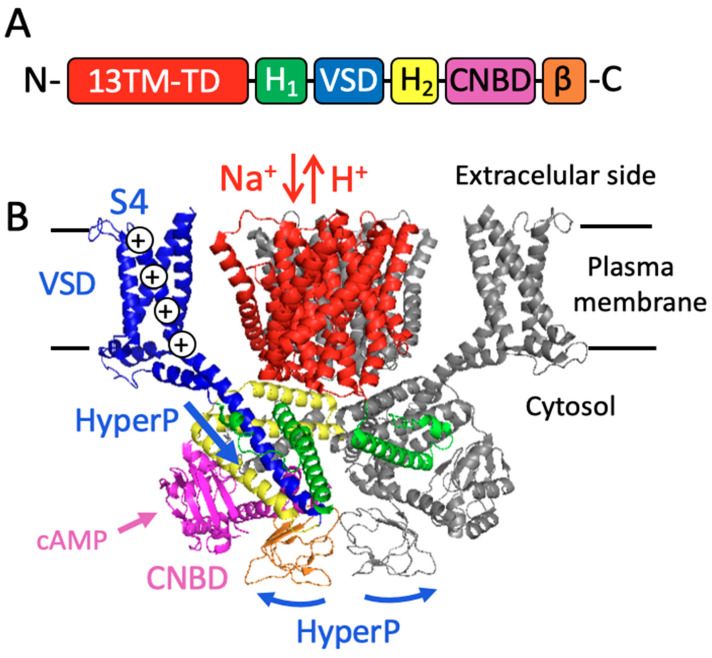
Structure of the sperm-specific Na^+^/H^+^ exchanger, sNHE (SLC9C). (**A**) depicts the domain arrangement of sNHE with color-coded features representing different domains: transporter domain composed of 13 TMs (13TM-TD, red), first cytosolic helices (H1, green), voltage sensor domain (VSD, blue), second cytosolic helices (H2, yellow), cyclic nucleotide-binding domain (CNBD, pink), and the C-terminal β strand domain (β, orange). (**B**) illustrates the 3D structure of an inactive state of sea urchin sNHE (*Sp*sNHE) dimer, determined by cryo-EM analysis (PDB ID: 8OTX, [76]). To highlight the interphase of the dimer in the cytosolic helix domain, one monomer is colored according to Scheme A, while the other monomer is represented in gray using the PyMOL program. Hyperpolarization (HyperP) of the membrane potential is expected to induce a downward movement of the positively charged S4 segment of the VSD, rendering the sNHE in an active state. Additionally, the binding of cAMP to the CNBD might facilitate the conformational change toward the active state.

Recent cryo-electron microscopy (Cryo-EM) analysis revealed that all animal NHEs (SLC9A1, SLC9A3, SLC9A9, SLC9B2, and SLC9C1) form a homodimer of conserved transporter domain (TD) composed of 13 transmembrane (TM) segments (13TM-TD) [97,98,99,100,101,102], as illustrated in red in Figure 2. The structure and function of the 13TM-TD of animal NHEs are like those of prokaryotic NHEs such as NapA and NhaP [103,104,105], whereas the NHE of *Escherichia coli* (NhaA) is composed of 12 TMs [106].

While all animal NHEs are anticipated to preserve a 13TM-TD with a similar electroneutral Na^+^/H^+^ exchange mechanism, it is noteworthy that the three groups (SLC9A, SLC9B, or SLC9C) exhibit distinct regulatory domains or regions at their N- or C-terminals.

The SLC9A group, encompassing NHE1-9, features a relatively large cytosolic region at the C-terminal functioning as an interaction and signaling hub, containing multiple regulatory sites, including a calcineurin B-homologous protein (CHP)-binding site, PI (4,5) P2-binding sites, Ca^2+^-calmodulin (CaM)-binding sites, and numerous phosphorylation sites (Pedersen and Counillon, 2019). Cryo-EM has also provided 3D structures of NHE1 and NHE3 in complexes with CHP1, which serves as an essential subunit of these NHE isoforms [100,101,107]. A recent proteomic analysis demonstrated the presence of NHE1 (SLC9A1) and CHP1 in human sperm [39].

In contrast to SLC9A, the SLC9B group (NHA1 and NHA2) possesses a small C-terminal cytosolic region. NHA2 has an additional N-terminal TM segment before the 13-TM transporter domain, displaying 14 TM segments with a disordered cytosolic region at the N-terminal [107]. In mice, both NHA1 and NHA2 were later immunodetected at the principal piece of the sperm flagellum (Figure 1). A double knock-out (KO) of NHA1 and NHA2 results in an infertile male phenotype with a deficiency in cAMP signaling and flagellar motility [57]. Interestingly, the infertility phenotype of the double-KO is similar to that observed in NHE10/sNHE-KO mice (SLC9C1). 

It is known that the zona pellucida (ZP) of the oocyte stimulates an increase in the pHi of homologous sperm in mammals [56,108]. In mice, NHA1 (SLC9B1) is proposed to be involved in this pHi increase, as sperm lacking NHA1 do not exhibit this response [56]. Although the involvement of a pertussis-toxin-sensitive G-protein in this process was reported [108], the interaction between the G-protein and NHA1 has not been fully demonstrated. Since NHAs (SLC9Bs) do not possess a well-defined regulatory domain at either their N- or C-terminus, their regulatory mechanism remains largely undisclosed. Further research is necessary to understand how NHAs are regulated, including their molecular interactions with other proteins and factors such as specific lipids.

In 2003, Garbers’ group reported a sperm-specific NHE (sNHE/NHE10, SLC9C1) that is essential for mice’s male fertility [16]. This protein is also important for human male fertility [18]. A unique feature of sNHE/NHE10 (SLC9C1) is the possession of a voltage-sensing domain (VSD), typically found in voltage-gated channels, after the 13-TM TD along with a cyclic nucleotide-binding domain (CNBD) at the C-terminal cytosolic region (Figure 2). The SLC9C group comprises SLC9C1 and SLC9C2. However, mouse sperm only expresses SLC9C1, located at the principal piece of the flagellum. In contrast, NHE11 (SLC9C2) was recently immunolocalized at the head of human sperm [58]. Unlike SLC9A and SLC9B, SLC9C is not conserved in some vertebrates such as amphibians, birds, and most ray-finned fishes, as well as in invertebrates like most insects, nematodes, and mollusks [109,110]. The distribution of SLC9C among the metazoans aligns well with those of sAC and CatSper, suggesting a functional coupling among these three proteins [26,110]. Indeed, it was demonstrated that sNHE physically associates with sAC, whose activity is diminished in sNHE-KO mouse sperm [22]. Despite the importance of sNHE/NHE10 in mouse sperm, establishing its functional expression in a heterologous system has not been achieved; therefore, its biochemical and biophysical characterization has been limited. Recently, we found that the VSD of mouse sNHE is toxic to bacteria because this domain forms a leaky ion channel [111], which may account for the technical challenges of studying it. Overcoming this technical hurdle promises to accelerate the characterization of this transporter.

The functional and structural characterization of SLC9C1 was advanced using the sea urchin ortholog (SpSLC9C1). Windler et al. (2018) expressed the sea urchin sNHE in Chinese hamster ovary (CHO) cells [91]. *Sp*SLC9C1 indeed functions as an NHE and is positively regulated by hyperpolarized membrane potential and cAMP through the VSD and CNBD, respectively. In 2023, two independent groups reported the 3D structure of SpSLC9C1 using cryo-EM [98,110]. As depicted in Figure 2B, *Sp*SLC9C1 forms homodimers. The positively charged fourth TM segment (S4) of the VSD has a long alpha helix, and its downward movement at negative potential is proposed to induce a conformational change of the cytosolic helix domain and the beta domain found at the C-terminal of SpSLC9C1, leading to an active state. Binding of cAMP to the CNBD induces its conformational change and is supposed to promote a downward movement of the S4 of the VSD. The AlphaFold Protein Structure Database (https://alphafold.ebi.ac.uk, accessed on 16 January 2024) predicts that mammalian SLC9C1 and SLC9C2 maintain a similar overall structure to that of *Sp*SLC9C1.

**Figure 3:**
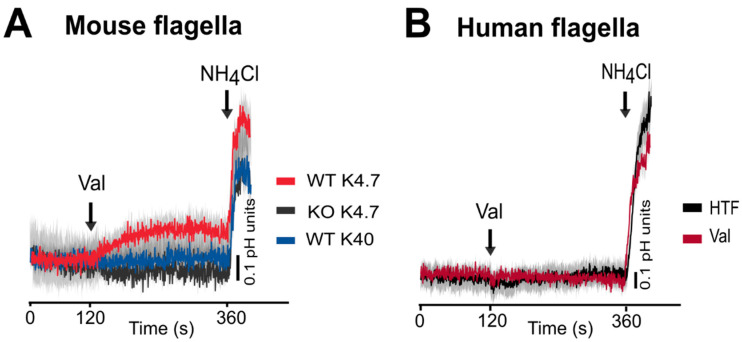
pHi changes in response to a valinomycin-induced hyperpolarization in mouse and human sperm. Sperm pHi was assessed using the pH-sensitive dual-emission fluorescence probe, SNARF-5F (Excitation= 530 nm; Emission= 575/640 nm) in single cell recordings. Panel (**A**) illustrates pHi changes in the midpiece of mouse sperm induced by 1 µM valinomycin (Val) followed by a 20 mM NH_4_Cl control addition. WTK4.7 (red trace) represents wild-type mouse sperm in a normal medium containing 4.7 mM K^+^, while KOK4.7 (black trace) indicates sperm from sNHE (SLC9C1) null mice in a normal medium. WTK40 (blue trace) indicates wild-type sperm in a medium with 40 mM K^+^. Panel (**B**) depicts pHi changes in the human sperm flagellar midpiece. HTF (black trace) indicates the addition of medium, serving as a negative control against the addition of 1 µM valinomycin (Val, red trace). The results are adapted from [18], with some modifications.

As the functional expression of mammalian SLC9C1 in a heterologous system has not been established, we characterized mouse and human SLC9C1 by measuring sperm pHi using the pH-sensitive probe SNAR-5F with a dual-emission imaging system [17,112]. A stimulus of hyperpolarization with valinomycin increases pHi in wild-type mouse sperm flagella but not in sNHE-KO mouse sperm (Figure 3A), supporting the idea that mouse sNHE/NHE10 (SLC9C1) functions similarly to the sea urchin ortholog. In contrast, the same stimulus did not increase pHi in human sperm (Figure 3B), demonstrating the species-specific function of this protein. Further studies on other mammalian sperm may help elucidate the structure-functional relationship of this protein. Interestingly, sNHE-KO mouse sperm exhibited a higher baseline pHi compared to wild-type sperm. This suggests that other mechanisms may compensate for the loss of sNHE/NHE10 (SLC9C1) function.

Recent human sperm proteomics indicated that SLC9B1 (NHA1), SLC9B2 (NHA2), SLC9C1 (sNHE/NHE10), and SLC9C2 (NHE11) were the predominant NHEs, along with a smaller amount of SLC9A1 (NHE1) [39]. While other NHEs may be present as minor components in human sperm, these five isoforms are expected to play important roles in sperm function. Additional research on these NHEs is essential to fully understanding how they regulate sperm pHi and this cell’s physiology.

### 2.4. The Voltage-Gated H^+^ Channel (Hv1)

Initial reports on voltage-dependent H^+^ currents date back to 1982 [113]. However, it was not until 2006 that the gene (HVCN1) and its corresponding protein (Hv1), responsible for this activity, were described [19,114]. Structurally, Hv1 channels exist as homodimers, with each subunit possessing only four TM segments (designated as S1 to S4). These segments serve dual roles as both the VSD and the H^+^ conducting pathway. In addition to the TM segments, Hv1 features cytosolic N- and C-terminal domains. The former contains several crucial amino acid residues that regulate its function, while the latter forms a coiled-coil structure, pivotal for regulating its dimerization [115].

Hv1 channels are highly H^+^ selective [116], and their activity is regulated, among other mechanisms, by the H^+^ gradient across the membrane. The greater the gradient, the lower the membrane potential required to induce an increase in channel conductance [117]. Upon activation, Hv1 channels facilitate outward H^+^ currents, leading to a notable pHi increase. This process is crucial for maintaining pHi homeostasis within the intracellular milieu. 

This protein is expressed in various tissues and cell types, and its involvement is significant in numerous physiological processes. One of the most studied is the respiratory burst in innate immune cells, where Hv1 seems to collaborate with NADPH oxidase to generate elevated levels of bactericidal reactive oxygen species, compensating for membrane depolarization and intracellular acidification, which are inadvertent side effects of NADPH oxidase enzymatic activity [118,119].

In 2010, the existence of outward H^+^ currents evoked by the Hv1 channel was reported in human sperm [20]. This protein was detected in the principal piece of human sperm flagellum [20,59], as well as in bull [120] and boar [121] sperm. Notably, no H^+^ current was observed in mouse sperm, indicating a species-specific distinction. Interestingly, a sperm-specific variant of Hv1, known as Hv1Sper, was found in humans [122]. These variants form functional dimers with full-length Hv1 monomers. One notable characteristic of Hv1Sper is its modified voltage-gating properties, leading to a shift in the activation curve towards more hyperpolarized voltages in the presence of pH gradients [122].

Hv1-evoked H^+^ currents were found to be heightened in capacitated sperm and inhibited by Zn^2+^, a well-known antagonist of this channel. Moreover, when human sperm were capacitated (in vitro) in the presence of Zn^2+^ or 5-Chloro Guanidine benzimidazole (Cl-GBI), another Hv1 antagonist, a pHi reduction was observed [50]. Notably, Hv1 antagonists impede flagellar hyperactivation [50] and the rolling swimming pattern in human sperm [59]. Similar effects on various kinetic parameters have been observed in both bull [120] and boar [121] sperm. Such effects on sperm motility can be explained by pHi acidification and the subsequent decrease in activity of the pHi-dependent Ca^2+^ channel CatSper [20,59]. Utilizing super-resolution microscopy, Miller and colleagues demonstrated that Hv1 is localized in between two of the CatSper longitudinal rows across the flagellum. The authors proposed that Hv1 forms a nano-localized pHi gradient essential for the asymmetrical activation of CatSper [59]. Evidence of this possible regulatory mechanism was obtained by Zhao and colleagues, who developed a peptide-based Hv1 inhibitor that diminishes the progesterone-induced [Ca^2+^]i increase in human sperm [60], a well-examined process dependent on CatSper activation [123].

These observations suggest that Hv1 plays a crucial role in maintaining the balance of H^+^ in human sperm, consequently impacting downstream processes regulated by pH, like CatSper channel activation. Though how this channel is activated during sperm capacitation is not fully understood, particularly considering that this process involves plasma membrane hyperpolarization [124]. Therefore, the positive Vm values necessary for Hv1 activation are unlikely to be achieved in sperm, at least in vitro. In this context, the short Hv1 variant, Hv1Sper (as discussed earlier), may be a possible explanation for this issue. Given the localization of Hv1 channels in the sperm principal piece, it became crucial to investigate whether mechanical stimuli generated by flagellar beating serve as a regulatory mechanism for channel activity. A tenfold higher Hv1 current is triggered upon mechanical stimulation in the presence of pH gradients, even at hyperpolarizing potentials (−30 mV) [125]. Additionally, it has been shown that albumin, a required component for in vitro capacitation, directly binds to Hv1, shifting the voltage threshold for channel activation to more negative potentials and enhancing the outward H^+^ current [61]. Furthermore, albumin also serves as a cholesterol acceptor, a critical process for promoting sperm capacitation, and acts as a molecule with an inhibitory effect on Hv1 activity [126]. According to this observation, albumin is significantly more abundant in uterine fluid (500 μM) compared to seminal fluid (15 μM), suggesting a potential involvement of this protein in the regulation of Hv1 during in vivo capacitation [61]. 

The cumulative evidence suggests a multimodal mechanism for Hv1 regulation in sperm. This mechanism involves both extracellular and intracellular cues, with their effects varying depending on the localization of the sperm within the female reproductive tract or the timing of capacitation.

**Figure 4:**
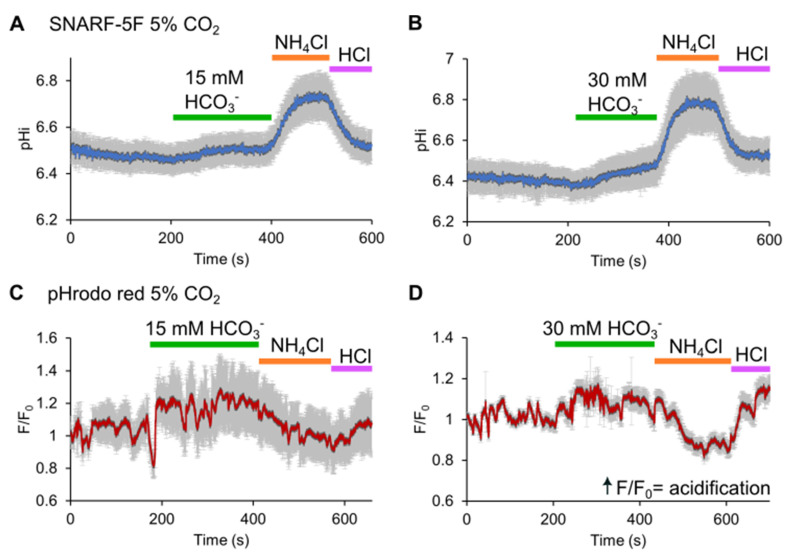
Indicator-dependent pHi changes in response to HCO_3_^−^ perfusion in human sperm. HCO_3_^−^ causes a pHi alkalinization in human sperm, as reported by the SNARF-5F dye, but a slight pHi acidification when pHrodo red is used. Representative pHi recordings using SNARF-5F (**A**,**B**) and pHrodo red (**C**,**D**) perfusing 15 or 30 mM HCO_3_^−^ (green rectangle) in a 5% CO_2_ environment. As positive controls, perfusions of 10 mM NH_4_Cl (orange rectangle) and 5 mM HCl (purple rectangle) are shown in each panel. Traces in each panel show average responses from 104 cells (for SNARF-5F) and 101 cells (for pHrodo red), with S.E.M. in gray. Ratiometric SNARF-5F measurements are reported as pHi values, whereas for pHrodo red, the F/F_0_ normalization is shown, and F = fluorescence intensity. ↑F/F_0_ indicates pHi acidification.

As discussed earlier, Grahn and colleagues propose that HCO_3_^−^ in the presence of 5% CO_2_, such as occurs in sperm capacitation, induces acidification instead of alkalization [39], contrary to previous reports without controlling CO_2_ [45,127,128]. Under CO_2_ equilibrium, Hv1 activation should occur only transiently and solely after a depolarizing stimulus, potentially serving as a mechanism to prevent excessive acidification caused by CO_2_ influx and subsequent HCO_3_^−^ production. This observation contrasts with previously discussed reports where Hv1 was suggested as a significant regulatory element of pHi during in vitro capacitation [20]. A potential source of variation between these reports may arise from differences in the timing of pHi measurements. Although in the study by Grahn et al. (2023) [39], the concentrations of HCO_3_^−^ and albumin used were consistent with previous studies, the longest recording was conducted after one hour of incubation in capacitation-promoting conditions. It is well known that many signaling pathways associated with capacitation require hours to unfold. To better understand these discrepancies, we performed pHi measurements using two pHi-sensitive dyes, SNARF-5F and pHrodo red, in a CO_2_ environment and perfusing HCO_3_^−^. Using SNARF-5F, we observed a pHi alkalinization during HCO_3_^−^ addition in both 15 and 30 mM HCO_3_^−^. This result is in accordance with previous studies (Figure 4). On the other hand, using pHrodo red, we found a slight pHi acidification after HCO_3_^−^ perfusion in a 5% CO_2_ environment. We believe that the simplest and most probable explanation for the differences between these pHi sensitive dyes is that SNARF-5F is a ratiometric dye, while pHrodo red is a single wavelength qualitative dye. Once the ratio between the SNARF-5F emissions is calculated, the noise caused by sperm movement is basically eliminated. This correction cannot be implemented with pHrodo red unless an additional dye is used. Previous studies demonstrated that adding HCO_3_^−^ elevates [Ca^2+^]i [129] and increases sperm flagellar beat frequency [130]. Therefore, it is likely that after HCO_3_^−^ addition, sperm flagellar beating intensifies and the pHrodo fluorescence measurements are altered.

Measuring pH in living cells is a challenging task. The strategies include direct determinations using H^+^-permeable microelectrodes or an indirect estimation using nuclear magnetic resonance of metabolites whose resonance frequency is influenced by pH. However, due to their versatility, pH-fluorescent-sensitive dyes are the most common approach to measuring pHi and pHa in sperm [131]. The Agilent Seahorse instrument measures mitochondrial activity in part by sensing extracellular acidification, which is also an indirect measurement of pHi. pH-sensitive and genetically modified proteins cannot be used in sperm since these cells are translationally silent. 

### 2.5. The Plasma Membrane Ca^2+^ ATPase (PMCA) Pump

The PMCA pump is a pivotal protein for [Ca^2+^]i homeostasis [132] that plays a vital role in Ca^2+^ clearance following an increase in [Ca^2+^]i in mouse sperm. Specifically, PMCA4 (ATP2B4), located in the principal piece of sperm flagella, is essential for sperm motility. Studies by Okunade [63] and Schuh [133] underscored the significance of PMCA4, as its absence leads to male infertility attributed to severe sperm motility deficiencies.

Although not widely recognized, the PMCA pump functions as a Ca^2+^/H^+^ exchanger powered by ATP [62]. Consequently, when it extrudes Ca^2+^ from the cell, pHi decreases. For instance, progesterone-induced increases in [Ca^2+^]i in human sperm, mediated by Ca^2+^ influx through CatSper channels [123], could be counteracted by PMCA4 extruding Ca^2+^ and indirectly by inactivating CatSper via pHi acidification at the sperm principal flagellum piece. As described in the Hv1 section, inhibition of this channel reduced the [Ca^2+^]i increase induced by progesterone in human sperm [60]. 

### 2.6. Monocarboxylate Transporters (MCTs/SLC16A)

Monocarboxylate transporters (MCTs) belonging to the SLC16A family are H^+^-linked transporters that facilitate the movement of pyruvate and lactate across the cell membrane [134,135]. To date, 14 genes have been classified within the SLC16A family; however, only four of these have been experimentally confirmed as MCTs: MCT1 (SLC16A1), MCT2 (SLC16A7), MCT3 (SLC16A8), and MCT4 (SLC16A3). The Michaelis constant (Km) values for lactate of MCT1 and MCT2 are relatively low, at 3–5 mM and 0.7 mM, respectively, indicating their significant role in lactate uptake into cells. Conversely, the Km value for MCT4 is comparatively high at 28 mM, suggesting its primary function in lactate extrusion from cells [135].

Although glycolysis is currently considered the predominant mechanism for ATP production-sustaining mammalian sperm flagellar beating, evidence shows that it can be maintained in a medium devoid of glucose if pyruvate or lactate are present [136,137]. Specifically, the presence of monocarboxylate transporters MCT1 and MCT2, along with the MCT-binding protein basigin/CD147 (BSG), has been confirmed in mouse sperm through Western blot and immunofluorescence techniques [64,65]. Furthermore, Mannowetz et al. (2012) [64] demonstrated that the addition of either D- or L-lactate lowers murine sperm pHi, with L-lactate having a more significant effect. This evidence supports the role of MCTs in facilitating lactate uptake and pHi acidification. 

On the other hand, mouse sperm possesses a testis-specific lactate dehydrogenase, lactate dehydrogenase C (LDHC), located in the principal piece of the flagellum [66]. This enzyme plays a crucial role by converting pyruvate into lactate and producing NAD^+^, a rate-limiting substrate for glycolysis. The absence of LDHC leads to male infertility, characterized by reduced ATP levels and decreased sperm motility. Interestingly, in mouse sperm suspensions supplemented with glucose as an energy source, LDHC-dependent lactate accumulation has been observed [66]. 

Recent human sperm proteomics [39] confirmed the presence of MCT1 (SLC16A1), basigin/CD147 (BSG), and MCT4 (SLC16A3). The presence of MCT4 suggests a role not only in lactate uptake but also in lactate extrusion, providing mechanisms for both acidification and alkalinization of pHi by MCTs. Additional work is necessary to deepen our understanding of the significance of MCTs in pHi regulation.

### 2.7. Acrosome pH (pHa) Regulation

The acrosome is a single exocytotic vesicle that covers a large part of the nucleus in the sperm head, forming a cap-shaped structure. It is a vesicle with an acidic pH (~5.5) [138,139,140], which also constitutes one of the main sperm Ca^2+^ stores [141,142]. The acrosome is derived from the Golgi apparatus and contains hydrolytic enzymes, which are proposed to facilitate sperm transit through the cumulus cell vicinity and the zona pellucida matrix to reach the oocyte [143,144]. However, some proposals consider that the acrosome is a lysosome-related organelle (LRO) [145,146]. LROs represent a family of membrane-covered organelles, present only in some specialized cells and known as melanosomes and lysosomes, among others [145].

pHa is maintained at acidic levels while sperm travel through the male reproductive tract [138]. After ejaculation and during capacitation in the female reproductive tract, a pHa increase occurs, allowing the activation of acrosomal enzymes and AR preparation. Nakanishi and colleagues (2001) [140] reported acrosomal alkalinization during capacitation in mouse sperm expressing a pH-dependent green fluorescent protein (EGFP) inside the acrosome. They estimated that before capacitation, pHa was 5.3 ± 0.1 and gradually increased to 6.2 ± 0.1 in capacitated cells [140]. Additionally, using Lysosensor green, a marker that partitions into acidic compartments, our group observed that the human sperm pHa becomes alkalinized after 6 h of incubation in a capacitating medium [67]. 

The maintenance of an acidic pHa and its subsequent alkalization during capacitation involves fine-tuning between pHa and pHi. The main transporters that regulate pHa will be described below.

### 2.8. The Vacuolar ATPase (V-ATPase)

The V-ATPase, or V1V0-ATPase, is a H^+^ pump located in the endomembrane system of all eukaryotic cells and is mainly responsible for acidifying the lumen of subcellular organelles such as lysosomes and endosomes [147,148]. The V-ATPase is essential for the maintenance of the luminal pH of these organelles, the ionic homeostasis of specialized tissues, protein trafficking, endocytosis, as well as the secretion of hormones and the release of neurotransmitters, among others [148,149]. The structure of the V-ATPase is like that of the F-ATP synthase found in the inner mitochondrial membranes of eukaryotic cells. The V-ATPase is a large enzymatic complex formed by multiple subunits, organized in two domains coupled by a rotating mechanism: the ATP hydrolysis domain (V1) and the H^+^ translocation domain (V0). The V1 domain is a 650 kDa complex located on the cytosolic side of the plasma membrane and is responsible for ATP hydrolysis [148,149,150]. It is composed of 8 different subunits named with capital letters (from A to H). The V0 domain is a 260 kDa complex that is embedded in the membrane and is responsible for the mobilization of H^+^ from the cytosolic to the lumen of an organelle or to the extracellular space. The V0 domain is made up of 6 subunits identified with lowercase letters (a, c, c′, c″, d, and e). All c subunits constitute proteolipid isoforms [148,149,150].

In mouse sperm, the V-ATPase has been immunolocalized in the acrosome [68]. Using anti-ATP6V1A antibodies, our group found this ATPase in the acrosome and midpiece of human sperm [67]. Confirming its location, bafilomycin A, a specific V-ATPase inhibitor, decreased the fluorescence intensity of Lysotracker, a marker of acidic pools, indicating acrosomal alkalinization [151]. Furthermore, bafilomycin A quickly alkalinizes the acrosome of capacitating sperm before this process advances [67]. Sun-Wada and colleagues [68] identified an isoform E1 of the V-ATPase specific to the testes and found in developing sperm. This isoform E1, originally named ATP6E1 and later renamed ATP6V1E2 (current nomenclature) [152], appears approximately when the sperm begin to mature and concentrate in the acrosome. This subunit is crucial for proper energy coupling between ATP hydrolysis and H^+^ pumping (acidification). Sun-Wada and co-workers [68] also observed that by disrupting ATP6V1E2 function, acrosomal acidification was affected, indicating that this isoform of the V-ATPase E subunit plays a vital role in acrosomal acidification during sperm development in mice [68]. Also, its homolog, originally designated as ATP6E2 (E2), now designated as ATP6V1E1, is present in testicular tissue but is ubiquitously expressed in other tissues [68,152]. These results suggest that the V-ATPase is important in the regulation of the mammalian sperm pHa.

In addition to the V-ATPase, the regulation and maintenance of pHa requires the flow of counterions (at least in other organelles), such that the electrochemical gradient (driving force generated by the difference in electrical charge and ion concentrations across the membrane) generated dissipates to maintain an acidic luminal pH. Anion influx must occur in parallel to the influx of H^+^. On the other hand, cation efflux through different channels or carriers has been proposed to contribute to the maintenance of pHa [153]. 

### 2.9. Role of Cl^−^ Channels in pHa Maintenance 

Cl^−^ influx regulates lysosomal acidification by providing counterions for H^+^ pumping [154]. Incubation of sperm in media lacking Cl^−^ inhibits most processes associated with capacitation: sperm do not hyperactivate, cannot undergo AR, and therefore cannot fertilize the oocyte [85,155]. Several types of Cl^−^ channels have been suggested to participate in sperm capacitation, such as ClCs [53,69], Ca^2+^-activated chloride channels (CaCC) [39,156], γ-aminobutyric acid (GABA) receptors, and glycine-activated receptors [157,158].

CaCCs of the TMEM16A type have been found in the plasma membrane of the human sperm head utilizing immunological and electrophysiological techniques [156]. This channel was proposed to participate in the AR [156]. In guinea pig sperm, TMEM16A inhibition by T16Ainh-A01 impaired capacitation, reduced progressive and hyperactivated motility, and blocked Pg-induced AR [159]. It is not yet known if TMEM16A participates in pHa regulation. TMEM16A, also called ANOCTAMINE1, was detected in proteomic studies in human sperm, together with another six ANOCTAMINES [39].

ClCs are a family of voltage-gated channels highly conserved in both prokaryotic and eukaryotic organisms [160]. This channel family is composed of 9 members, of which ClC-3 is a voltage-dependent intracellular electrogenic 2Cl^−^/H^+^ exchanger. ClC-3 plays important roles in volume regulation and sperm motility and was detected in the midpiece of human sperm [69]. However, how these channels regulate pHa at the level of the acrosomal membrane is still not clear.

Functional CFTR was documented in mouse testicular sperm in patch clamp experiments, which were corroborated by the fact that Cl^−^ current stimulation by cAMP and sensitivity to inhibitor CFTRinh-172 were absent in sperm from ΔF508-CFTR mice [86]. CFTR channel inhibition also blocks the acrosome alkalinization that occurs during human sperm capacitation, indicating it modulates pHa in human sperm, probably by regulating HCO_3_^−^ entry [67]. Further research is required to understand the role of CFTR in the regulation of pHa.

### 2.10. Role of [Ca^2+^]i in pHa Regulation

The Ca^2+^ dependence of H^+^ uptake and maintenance in acidic organelles indicates a closely coupled homeostatic system. It is known that, as in the endoplasmic reticulum (ER), organelles such as lysosomes, lysosome-related organelles, secretory vesicles, vacuoles, and acidocalcisomes express Ca^2+^-permeable channels that can participate in intracellular signaling processes. These include members of the transient receptor (TRP) ion channel family, two-pore channels (TPC), ATP-activated ionotropic receptors (P2X), inositol triphosphate (IP3), and ryanodine (Ryr) receptors [161,162]. In mammalian sperm, acrosomal Ca^2+^ concentration is regulated by the IP3R and ryanodine receptors [163], the sarco/endoplasmic reticulum Ca^2+−^ATPases (SERCA) [164], and the two-pore channel 1 (TPC1) [162,165]. TPC1 channels participate in the acrosomal Ca^2+^ release regulated by pHa [162].

Though much is known about sperm [Ca^2+^]i regulation, how this cation modulates pHa lacks detail. The importance of [Ca^2+^]i for pHa regulation is manifested by the delay pHa alkalinization undergoes when human sperm are capacitated in a nominal Ca^2+^-free medium [67]. In mice and human sperm, acrosomal alkalinization elevates [Ca^2+^]i and triggers AR [166]. At least in mouse sperm, pHa alkalinization is important for acrosomal Ca^2+^ efflux and extracellular Ca^2+^ influx during AR [162].

IP3Rs are expressed in most animal cells and are responsible for the release of Ca^2+^ from intracellular Ca^2+^ stores; for instance, in the ER [167,168] and the Golgi apparatus [169]. These channels are also present in other intracellular organelles and participate in cell signaling processes. The IP3R has been detected in the acrosomes of several mammalian sperm species [168], but also in their midpiece and cytoplasmic droplets [168,170]. IP3Rs have been postulated to participate in the regulation of [Ca^2+^]i oscillations [171]. In non-capacitated human spermatozoa, the presence of [Ca^2+^]i oscillations in the sperm head is associated with AR inhibition [172,173]. Interestingly, [Ca^2+^]i oscillations are more frequent at a more acidic pHi (6.5) and decrease at a neutral or alkaline pH (7.4–8), the latter being correlated with greater AR induced by progesterone [173]. How pHe modulates pHi and pHa and their interrelation with [Ca^2+^]i remains to be fully elucidated.

### 2.11. Role of HCO_3_^−^ in pHa Regulation

Sperm capacitation requires elevation of cAMP, pHi, and [Ca^2+^]i, which are modulated by the entry of HCO_3_^−^ [6,23]. The participation of HCO_3_^−^ in the regulation of pHa has been evaluated mainly in mice and humans. Incubation of human sperm in a medium without HCO_3_^−^ inhibits the capacitation-dependent acrosome alkalinization, and therefore the acrosome remains acidic [67]. Interestingly, inhibiting CFTR (CFTR inh-172), which is known to participate in the initial entry of HCO_3_^−^ associated with capacitation [51], also blocked the acrosome alkalinization. These results suggest that CFTR could be important in the regulation of the HCO_3_^−^ influx required for acrosome alkalinization during capacitation. Similar results were observed in mice when sperm were incubated with DIDS, a Cl^−^/HCO_3_^−^ exchanger inhibitor. DIDS completely blocked acrosome alkalinization, suggesting that a Cl^−^/HCO_3_^−^ exchanger contributes to pHa regulation during capacitation [140]. These exchangers are considered important in the sustained entry of HCO_3_^−^, which helps integrate the sequence of changes associated with sperm capacitation [75]. The Cl^−^/HCO_3_^−^ exchanger (band 3, AE1 or SLC4A1) has been localized using immunohistochemistry in the equatorial segment of the human and rat sperm heads, forming a ring-like structure around which are organic compounds that are derived from ammonia and can act as the head close to or at the plasma membrane [36]. These results provide evidence that HCO_3_^−^ contributes significantly to the maintenance of pHa and acrosome alkalinization during capacitation.

It has recently been proposed that the activity of CAs in the conversion of CO_2_ into HCO_3_^−^, has great relevance for the regulation of pHi [39]. However, the role of these enzymes in the regulation of pHa has not been studied. Interestingly, mouse sperm acrosome proteomic studies identified different CAs (CA1, CA2, CA3, and CA4) [174].

### 2.12. Role of Na^+^/H^+^ Exchanger (NHE) in pHa Regulation

Few studies have dealt with the presence, distribution, and function of intracellular NHEs in mature sperm, especially in the acrosome. SLC9C2 (NHE11) was recently found in the sperm head, at the level of the plasma membrane that covers the acrosome, in both rat and human sperm [58]. However, its exact function is not yet known, although it is presumed that it may regulate AR or the sperm-egg fusion process [58].

## 3. Role of pHa during Sperm Mammalian Capacitation

An increase in pHa occurs in mouse sperm during capacitation, and it is proposed that this alkalinization can activate intra-acrosomal enzymes and stimulate spontaneous AR [139,140,143,166]. In human sperm, pHa also increases progressively during capacitation [67]. Interestingly, CatSper inhibitors such as mibefradil and NNC55-0396, at micromolar concentrations, not only induce Ca^2+^ influx but also acrosomal Ca^2+^ release and AR by increasing pHa in mouse and human sperm. Similar results were obtained in a nominally Ca^2+^-free medium [166]. These inhibitors are secondary permeable amines, which are organic compounds that can permeate into acidic compartments, elevating their luminal pH [175]. All this suggests that alkalinization of the acrosome is an essential step for the release of Ca^2+^ from this compartment and consequently for the AR [166]. Furthermore, it has been shown that pHa elevation promotes the disaggregation of the amyloid acrosomal matrix, favoring the exocytosis of its contents [176,177]. Our findings do support the idea that acrosomal alkalinization may swell the acrosome, induce acrosomal Ca^2+^ release, and promote outer acrosomal membrane fusion with the plasma membrane [67,166]. Recently, it was demonstrated that pHa alkalinization stimulates TPC1 channels that mediate acrosomal Ca^2+^ release and that the osmotic component plays a minor role in this response. In turn, acrosomal Ca^2+^ efflux would activate plasma membrane Ca^2+^ CRAC-type channels to favor the AR process [162]. pHa may serve as a marker of capacitation status, reflecting sperm readiness to respond to fertilization triggers.

**Figure 5:**
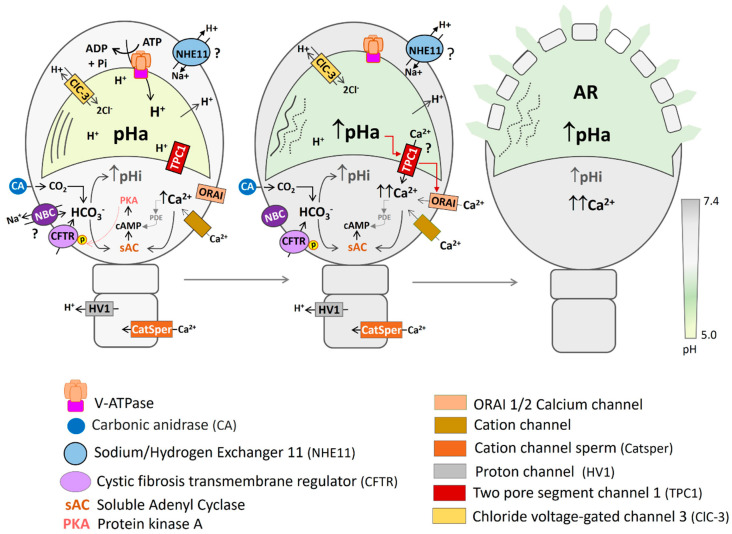
Model of the molecular entities that regulate pHa in human sperm. Under non-capacitated conditions (NC), the pHa is acidic, due mainly to the active pumping of H^+^ mediated by the V-ATPase into the acrosomal lumen and the flow of counterions through transport such as ClC-3. As capacitation initiates, HCO_3_^−^ enters the cell through different channels and transporters, and/or it is produced inside by the conversion of CO_2_, H_2_O, and H^+^. sAC is stimulated by HCO_3_^−^, elevating cAMP levels and activating PKA, allowing the phosphorylation of several proteins, including CFTR channels, which also may allow the entry of HCO_3_^−^. During capacitation, pHi also increases, favoring Ca^2+^ influx, which also enhances sAC activity. V-ATPase allows the acrosome to remain acidic during the first hours of capacitation. The continuous entry of HCO_3_^−^, as well as the exit of H^+^ from the cytosol, through the Hv1 channel in the case of human sperm or through NHEs in other mammals, stabilizes the cytosolic alkalinization, dissipates the H^+^ gradient, decreases V-ATPase activity, and induces the alkalinization of the acrosome. Other mechanisms, not yet described, could also regulate the activity of the V-ATPase. The pHa increase destabilizes the acrosomal matrix, producing acrosome swelling and probably TPC1 channel activation, releasing acrosomal Ca^2+,^ which in turn stimulates extracellular Ca^2+^ uptake through ORAI channels (1 and 2). Both acrosome alkalinization and [Ca^2+^]i increases induce AR. Arrow indicates increase of the ion (↑, ↑↑). We place the sign (?) to highlight that some transporters or channels, although they have been detected, their exact location and identity has not been fully established (NBC), or their function in humans is unknown (TPC1, NHE11).

The evidence described previously suggests that prior to capacitation the acrosome remains acidic (~5.5) due, at least in part, to active V-ATPase pumping coupled to counterion flow (i.e., Ca^2+^, Cl^−^). In human sperm, it is proposed that upon initiation of capacitation, HCO_3_^−^ uptake through several molecular entities increases sperm pHi and stimulates sAC, leading to elevated cAMP levels that activate PKA. Additionally, CO_2_ enters the sperm by diffusion through the cell membrane. CAs activity facilitates the conversion of CO_2_, H_2_O, and H^+^ into HCO_3_^−^, playing a role in the general CO_2_/HCO_3_^−^/H^+^ balance and contributing to pHi regulation and sAC activation. Ca^2+^ influx, via CatSper and other channels, also stimulates sAC during capacitation. The pHa remains acidic during the first hours of capacitation due to the action of the V-ATPase and other mechanisms that we do not yet know. The entry of HCO_3_^−^ is maintained over time, stabilizing cytosolic alkalinization and maintaining the exit of H^+^ from the cell, modifying the cytosol-acrosome H^+^ gradient. Dissipation of the acrosomal H^+^ gradient and/or inactivation of the V-ATPase induce alkalinization of the acrosome [67]. On the other hand, the increase in pHa stimulates TPC1 channels, promoting acrosomal Ca^2+^ release, which induces extracellular Ca^2+^ influx through Ca^2+^ release activated Ca^2+^ channels (CRACs) to finally induce AR [162] (Figure 5). 

Although the spatial and temporal organization of the different mechanisms that regulate pHa is still not fully defined, the current model of pHa regulation in mammalian spermatozoa offers an overview of the mechanisms involved in this process. Further research is needed to advance our understanding of the complex signaling network that regulates sperm pHa and its interconnection with the regulatory pathways shared with pHi.

## 4. Perspectives

In this review, we have emphasized the importance of sperm pH regulation, which is complex and distinct for swimming, capacitation, and the AR. pHi alkalinization is recognized as an important capacitation marker and has been proposed recently as a tool to help fertility clinics predict fertilization success when using in vitro fertilization (IVF) or intracytoplasmic sperm injection (ICSI) procedures [50,178].

We have attempted to outline the transport elements involved in these functions and give a glimpse of how they may be orchestrated. What we have at present is a sketch, and therefore much research is needed to begin to envision the complete set of players and the choreography of how cytosolic and organellar pH are regulated in each sperm function. 

HCO_3_^−^ is one of the central factors for mammalian sperm capacitation. Therefore, its transmembrane transporters, such as CFTR and the SLC4 family proteins, as well as CAs, have been considered important for mammalian sperm pHi regulation. However, at least two human sperm proteomic reports did not detect CFTR or any SLC4 family proteins [39,179]. Though these results do not necessarily imply the absence of these proteins in human sperm due to the sensitivity limitations of the proteomic approach [180,181], they question the role of CFTR and other SLC4 transporters in human sperm physiology. In addition, pHi measurements in this latter work indicated that the addition of external HCO_3_^−^ decreased the pHi of human sperm instead of increasing it, as previously reported [45,112,127] and shown here. This result suggests that permeation of CO_2_ through the sperm plasma membrane is more significant than HCO_3_^−^ influx through its transmembrane transporters, questioning their relevance, a notion that must be further investigated. Clarification of these opposing results is of outmost importance for sperm function and specific ion channel regulation by alkalinization. This is particularly relevant considering the initial HCO_3_^−^ stimulus that occurs in the epididymis and afterwards in the female tract have been established as triggers to control and promote fertilization. The regulation of pHi involves a large set of proteins whose activity may change during sperm transit in the female tract. Most pHi measurements in the laboratory represent only a snapshot of this parameter under conditions that may contribute to such discrepancies. Although it is generally believed that a H^+^ gradient dissipates immediately through an H^+^ jump, a depletion of this cation near the Hv1 channel has been reported during channel activation, even in the presence of a 10 mM pH buffer [182]. This observation suggests that the nano-scale arrangement between pH modulators and their effectors is a significant issue to address. Considering recent advances in super resolution microscopy [183], cryo-electron microscopy [184,185], and cryo-electron tomography [186], future work will reveal how nanoscopic complexes regulate local sites to achieve the required pHi and [Ca^2+^]i responses to control the flagellar beat, the AR, and fertilization.

The acrosome becomes alkalinized during mammalian sperm capacitation in a manner dependent on the presence of HCO_3_^−^ and Ca^2+^ in the external medium. The regulation of the V-ATPase and how it is influenced by the associated ACs-PKA signaling pathway is yet to be established. The impact of pHa on sperm physiology is only beginning to be understood. It is proposed that the pHa increase is a key step in initiating AR. The specific ion transporters and proteins responsible for pHa regulation are not fully characterized, and their relative contribution to acrosome alkalinization in triggering this reaction under physiological conditions needs further elucidation. It is also relevant to know what other transporters and/or channels are present in the acrosome that can directly or indirectly modulate pHa and determine its physiological influence on fertility.

Notably, questions that seemed answered have re-emerged [108,187,188], and new ones have been unveiled thanks to technological and computational (AI) advances. We now must determine which are the physiological agonists of the AR, what is the identity of their receptors, and how they are regulated. What is the variability of these components in each species? Is the “spontaneous AR” orchestrated and relevant for the AR and fertilization? What is the role of the pHi increase associated with the AR, and how is it triggered and regulated? Why is the AR-related pHi elevation G protein dependent? How are pHi and pHa interrelated and coordinated during capacitation and the AR?

## Figures and Tables

**Table 1:** Proteins related to pHi regulation in mammalian sperm.

Molecule	Species	Cellular Localization	Related Function	Reference
CAI	Hs	PP	Control of motility, participation in the RA, HCO_3_^−^/CO_2_ balance and pH regulation	[34,35]
CAII	Hs; Mm	PP		[34,35,36,41]
CAIV	Hs; Mm	MP		[35,37]
CAXIII	Hs	MP		[35,42]
AE1 (SLC4A2)	Hs	H; PP	Its participation in pH regulation has not been determined.	[43]
AE2 (SLC4A2)	Mm	N.D.	Mice lacking the expression of this transporter present infertility problems, due to a failure in spermatogenesis. Its participation in pH regulation has not been determined.	[44]
NBCe1 (SLC4A4)	Hs; Mm	N.D.	Functional experiments suggested a role in membrane hyperpolarization and HCO_3_^−^ transport.	[45]
NBCe2 (SLC4A5)	Hs; Mm	N.D.		
DRA (SLC26A3)	Hs; Mm	MP	Role in membrane hyperpolarization, HCO_3_^−^ transport and pH regulation of mouse sperm	[39,46,47,48]
PAT1 (SLC26A6)	Hs; Mm	MP		
CFTR	Hs; Mm	Eq; MP	Heterozygous CFTR mutant mice showed lowered fertility rates. Pharmacological inhibition affects capacitation related events including intracellular alkalization	[46,49,50,51,52,53]
NHE1 (SLC9A1)	Hs; Mm	MP	Possible participation in plasma membrane hyperpolarization	[54,55]
NHE5 (SLC9A5)	Hs; Mm	MP	N.D.	[55]
NHA1 (SLC9B1)	Mm	PP	A double knock-out (KO) of NHA1 and NHA2 results in an infertile male phenotype with a deficiency in cAMP signaling and flagellar motility	[56,57]
NHA2 (SLC9B2)	Mm	PP		
sNHE/NHE10 (SLC9C1)	Hs; Mm	PP	Essential for male fertility in both humans and mice.	[16,17,18,22,56,57,58]
sNHE/NHE11 (SLC9C2)	Hs	PP	N.D.	
Hv1	Hs	PP	Regulates human sperm pH. Participation in hyperactivation and in CatSper-dependent intracellular calcium increase.	[20,50,59,60,61]
PMCA4 (ATP2B4)	Mm	PP	Knockout mouse is infertile. PMCA pump functions as a Ca^2+^/H+ exchanger powered by ATP	[62,63]
MCT1 (SLC16A1)	Mm	MP; PP	Facilitate lactate uptake and evokes pHi acidification.	[64,65]
MCT2 (SLC16A7)	Mm			
LDHC	Mm	PP	Converting pyruvate into lactate and producing NAD+ and consuming protons. Knockout mouse is infertile.	[66]
v-ATPase	Hs; Mm	Ac	Regulates intra acrosomal pH. Essential for acrosome reaction	[67,68]
ClC3	Hs	Ac	Volume modulation. Possible participation in intra acrosomal pH regulation	[69]

Abbreviations: Hs—Human (Homo sapiens); Mm—Mouse (Mus musculus); Ac—Acrosomal; Eq—Equatorial segment; H—Head; MP—Midpiece; PP—Principal piece; N.D.—non-determined.
